# Interferon Alpha Induces Sustained Changes in NK Cell Responsiveness to Hepatitis B Viral Load Suppression In Vivo

**DOI:** 10.1371/journal.ppat.1005788

**Published:** 2016-08-03

**Authors:** Upkar S. Gill, Dimitra Peppa, Lorenzo Micco, Harsimran D. Singh, Ivana Carey, Graham R. Foster, Mala K. Maini, Patrick T. F. Kennedy

**Affiliations:** 1 Hepatology, Centre for Immunobiology, Blizard Institute, Barts and The London School of Medicine & Dentistry, QMUL, London, United Kingdom; 2 Department of Hepatology, The Royal London Hospital, Barts Health NHS Trust, London, United Kingdom; 3 Division of Infection & Immunity, UCL, London, United Kingdom; 4 Institute of Liver Studies, Kings College Hospital, London, United Kingdom; Albany Medical College, UNITED STATES

## Abstract

NK cells are important antiviral effectors, highly enriched in the liver, with the potential to regulate immunopathogenesis in persistent viral infections. Here we examined whether changes in the NK pool are induced when patients with eAg-positive CHB are ‘primed’ with PegIFNα and importantly, whether these changes are sustained or further modulated long-term after switching to nucleos(t)ides (sequential NUC therapy), an approach currently tested in the clinic. Longitudinal sampling of a prospectively recruited cohort of patients with eAg+CHB showed that the cumulative expansion of CD56^bright^ NK cells driven by 48-weeks of PegIFNα was maintained at higher than baseline levels throughout the subsequent 9 months of sequential NUCs. Unexpectedly, PegIFNα-expanded NK cells showed further augmentation in their expression of the activating NK cell receptors NKp30 and NKp46 during sequential NUCs. The expansion in proliferating, functional NK cells was more pronounced following sequential NUCs than in comparison cohorts of patients treated with de novo NUCs or PegIFNα only. Reduction in circulating HBsAg concentrations, a key goal in the path towards functional cure of CHB, was only achieved in those patients with enhancement of NK cell IFNγ and cytotoxicity but decrease in their expression of the death ligand TRAIL. In summary, we conclude that PegIFNα priming can expand a population of functional NK cells with an altered responsiveness to subsequent antiviral suppression by NUCs. Patients on sequential NUCs with a distinct NK cell profile show a decline in HBsAg, providing mechanistic insights for the further optimisation of treatment strategies to achieve sustained responses in CHB.

## Author Summary

Current therapies for CHB are limited in achieving HBsAg decline and loss leading to a cure. Although PegIFNα may be used, the majority of patients progress to NUC therapy due to treatment failure. PegIFNα and NUCs used in isolation act differentially on the immune response; PegIFNα induces NK cell activation and NUC therapy may partially restore T cell function. Data, however, are limited on the immune effects when these therapies are used in sequence or in combination. Here, we analysed the immune effects of PegIFNα followed by sequential NUC therapy and show this treatment strategy maintains the cumulative expansion of antiviral CD56^bright^ NK cells, following PegIFNα-priming. HBsAg reduction was greater in patients treated with sequential NUCs when compared with de novo NUCs, highlighting the potential benefit of PegIFNα-priming. Such sustained boosting of NK cells on sequential NUCs following PegIFNα-priming has not previously been described, raising the potential of ‘long-lived’ NK cell populations in keeping with their emerging adaptive features. These findings provide a mechanistic and immunological rationale to explore this treatment strategy for CHB whilst awaiting the emergence of new therapies in the field.

## Introduction

Chronic Hepatitis B (CHB) related cirrhosis and hepatocellular carcinoma (HCC) account for approximately 600,000 deaths per year [1]. Current treatments for Hepatitis B virus (HBV) include Pegylated Interferon-Alpha (PegIFNα) and nuleos(t)ide analogues (NUCs). Although PegIFNα provides higher rates of off-treatment HBsAg loss, the gold standard treatment endpoint, [2,3] this is observed in a small proportion of patients. Alternatively, NUCs require lifelong administration to maintain long-term viral suppression and HBsAg loss as a treatment endpoint is sub-optimal [4–6]. These poor treatment outcomes highlight the limitations of current licensed therapies used in isolation. This is the impetus for the exploration of combination or sequential therapy strategies to improve treatment endpoints, [7,8] and importantly provide an immunological and mechanistic rationale to guide future therapeutic strategies.

The hallmark of CHB is a dysfunctional immune response; the CD8 T cell repertoire displays an exhausted phenotype, [9,10] and similarly the antiviral potential of NK cells is also impaired [11]. NK cells are important innate effector cells making up a significant proportion of the intrahepatic infiltrate. We have previously demonstrated that the immunoregulatory CD56^bright^ NK cell subset is highly enriched in the HBV-infected liver, expressing TNF-related apoptosis-inducing ligand (TRAIL) [12]. Their ability to produce cytokines (IFNγ) allowing non-cytolytic clearance of HBV-infected hepatocytes has also been shown to be impaired in CHB [11]. IFNα potently activates NK cells and we recently demonstrated that PegIFNα therapy in eAg negative disease led to a dramatic expansion of activated CD56^bright^ NK cells with enhanced antiviral potential, though this effect reduced on treatment cessation [13]. Notably NUC treated eAg negative patients did not show similar NK cell boosting [11] but conversely demonstrated partial restoration of HBV-specific T cells [14,15].

Here we investigated whether PegIFNα was able to mediate a similarly potent expansion of functional NK cells in eAg positive CHB as noted in eAg negative disease, and whether any such boosting could be maintained in patients progressing to sequential NUC therapy following PegIFNα. Longitudinal on-treatment NK cell responses were analysed throughout the course of PegIFNα+/- sequential NUCs, and correlated with clinical parameters of treatment response. We report for the first time that functional NK cell responses are restored, upon *in vivo* administration of PegIFNα, in eAg positive CHB and importantly these effects are preserved on sequential NUCs, with an associated decline in quantitative HBsAg exceeding that seen with either de novo NUC or PegIFNα therapy alone [16]. Insights into this mechanism of innate boosting in patients receiving sequential NUCs provides further scientific rationale to support re-evaluation of future treatment strategies.

## Results

### Expanded CD56^bright^ NK cells are maintained on sequential NUC therapy

**Fig 1:**
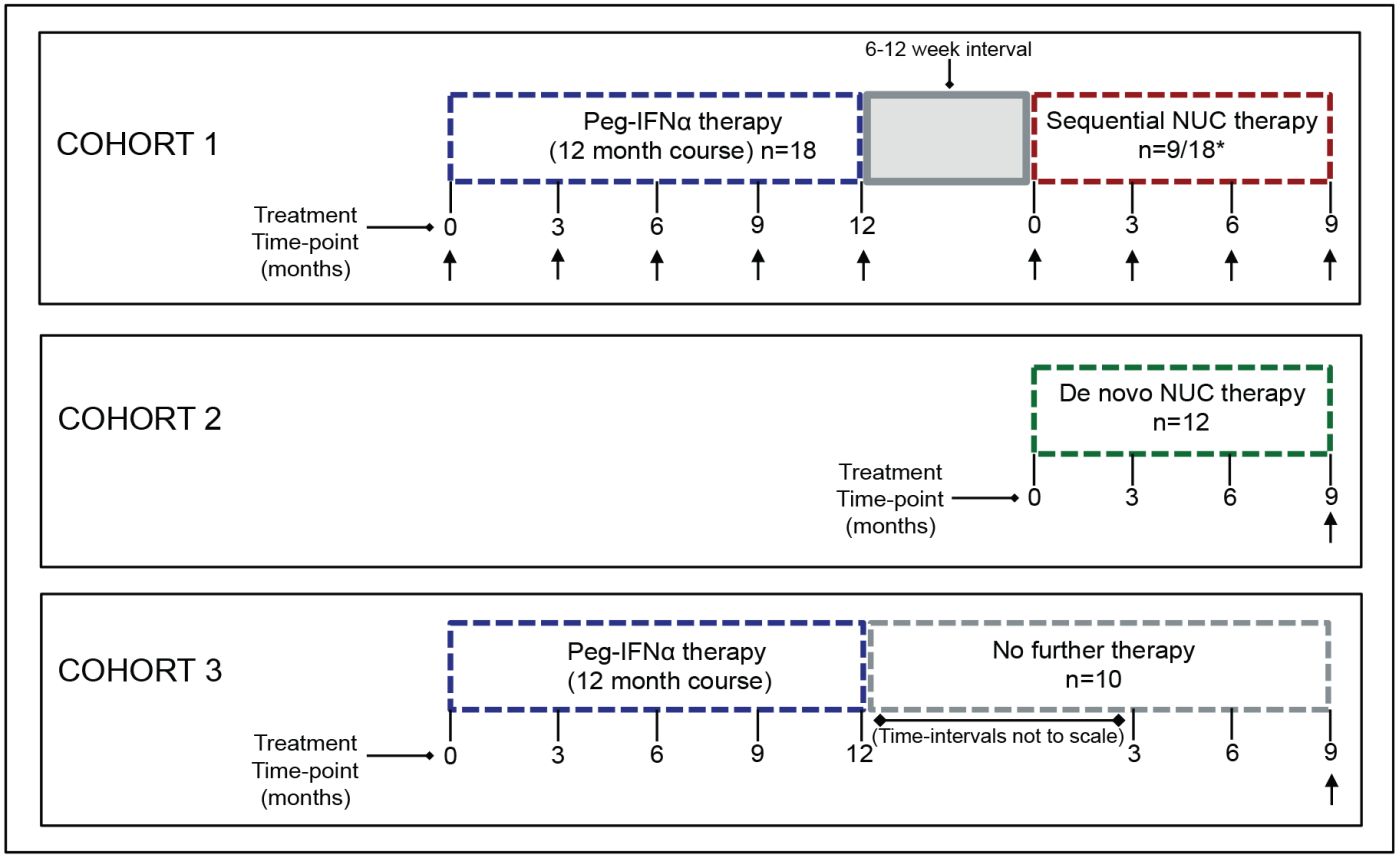
Schematic overview of patients studied for immune analysis. Cohort 1 indicates sequential NUC therapy patients studied; 18 consecutive patients were analysed longitudinally during a 48 week course of PegIFNα therapy (blue dashed outline), of which 9/18 patients progressed to sequential NUC therapy, following a 6–12 week interval gap (grey shaded box), and were sampled longitudinally; *indicates 5 further patients analysed undergoing sequential NUC therapy, sampled at time-point month 0 and 9 only on sequential therapy (total sequential NUC therapy cohort; n = 14) (red dashed outline). Cohort 2; n = 12 patients analysed at a single time-point at viral suppression on de novo NUC therapy (green dashed outline). Cohort 3; n = 10 patients treated with PegIFNα therapy for 48 weeks that did not undergo any further treatment (grey dashed outline), and sampled at 9 months following cessation of PegIFNα. Arrows under time-points indicate sampling time for immune analysis in each cohort.

We analysed NK cell subsets in patients, pre, during and following the cessation of PegIFNα therapy; 9/18 patients, consecutively studied, progressed to sequential NUC therapy and were studied longitudinally with 3-monthly sampling until viral suppression was achieved. A further 5 patients were studied cross-sectionally during sequential NUCs (Table 1, S1 Table, Fig 1).

**Table 1:** Baseline characteristics & clinical features of patients (Cohort 1).

	Age	Gender	Genotype	ALT	HBV DNA	HBsAg	Fibrosis	Outcome	Sequential Therapy
				(IU/L)	(log_10_IU/ml)	(log_10_IU/ml)	(Ishak)		
	[32]			[99]	[7.54]	[4.31]			
Pt.1	35	M	A	132	3.68	3.37	2	Responder* ^ ^¶^	No
Pt.2	49	M	C	68	4.36	4.49	3	Non-responder	Yes
Pt.3	32	M	C	57	5.74	3.73	3	Non-responder	Yes
Pt.4	38	F	B	52	8.51	4.50	1	Non-responder	Yes
Pt.5	26	M	C	62	7.21	3.85	4	Responder*	No
Pt.6	29	M	C	115	8.34	4.80	1	Non-responder	Yes
Pt.7	26	F	D	29	9.06	4.47	ND	Responder* ^¶^	No
Pt.8	26	F	C	60	8.28	3.86	2	Non-responder	Yes
Pt.9	32	M	C	85	9.16	5.04	1	Non-responder^¶^	No
Pt.10	37	F	B	341	8.11	3.02	1	Responder* ^¶^	No
Pt.11	35	M	D	91	6.80	4.53	1	Non-responder	Yes
Pt.12	22	M	A	187	8.20	4.47	3	Non-responder^¶^	No
Pt.13	32	M	C	51	8.69	4.07	2	Non-responder	Yes
Pt.14	31	F	E	55	7.30	4.33	1	Non-responder^¶^	No
Pt.15	38	F	B	102	7.96	4.17	ND	Non-responder	Yes
Pt.16	34	M	D	133	8.03	4.24	5	Non-responder	Yes
Pt.17	32	F	D	40	4.46	4.46	1	Non-responder^¶^	No
Pt.18	26	F	D	63	8.24	4.68	2	Non-responder^¶^	No
Pt.19^§^	55	M	A	95	8.37	5.10	ND	Non-responder	Yes
Pt.20^§^	18	M	A	86	8.18	4.31	3	Non-responder	Yes
Pt.21^§^	30	M	C	112	7.98	4.54	1	Non-responder	Yes
Pt.22^§^	40	M	A	167	9.25	4.52	ND	Non-responder	Yes
Pt.23^§^	33	M	D	99	7.48	4.50	1	Non-responder	Yes

- Numbers in brackets under headings; age = median values; ALT, HBV DNA & HBsAg = mean values

- ND: Test not done

- *sustained HBeAg seroconversion & HBV DNA <2000IU/ml (<3.30 logIU/ml) 6-months post cessation of Peg-IFNα

- ^sustained HBsAg loss

- ^¶^lost to follow-up/refused sequential NUC therapy

- ^§^sampled only at selected time-points on sequential NUC therapy

**Fig 2:**
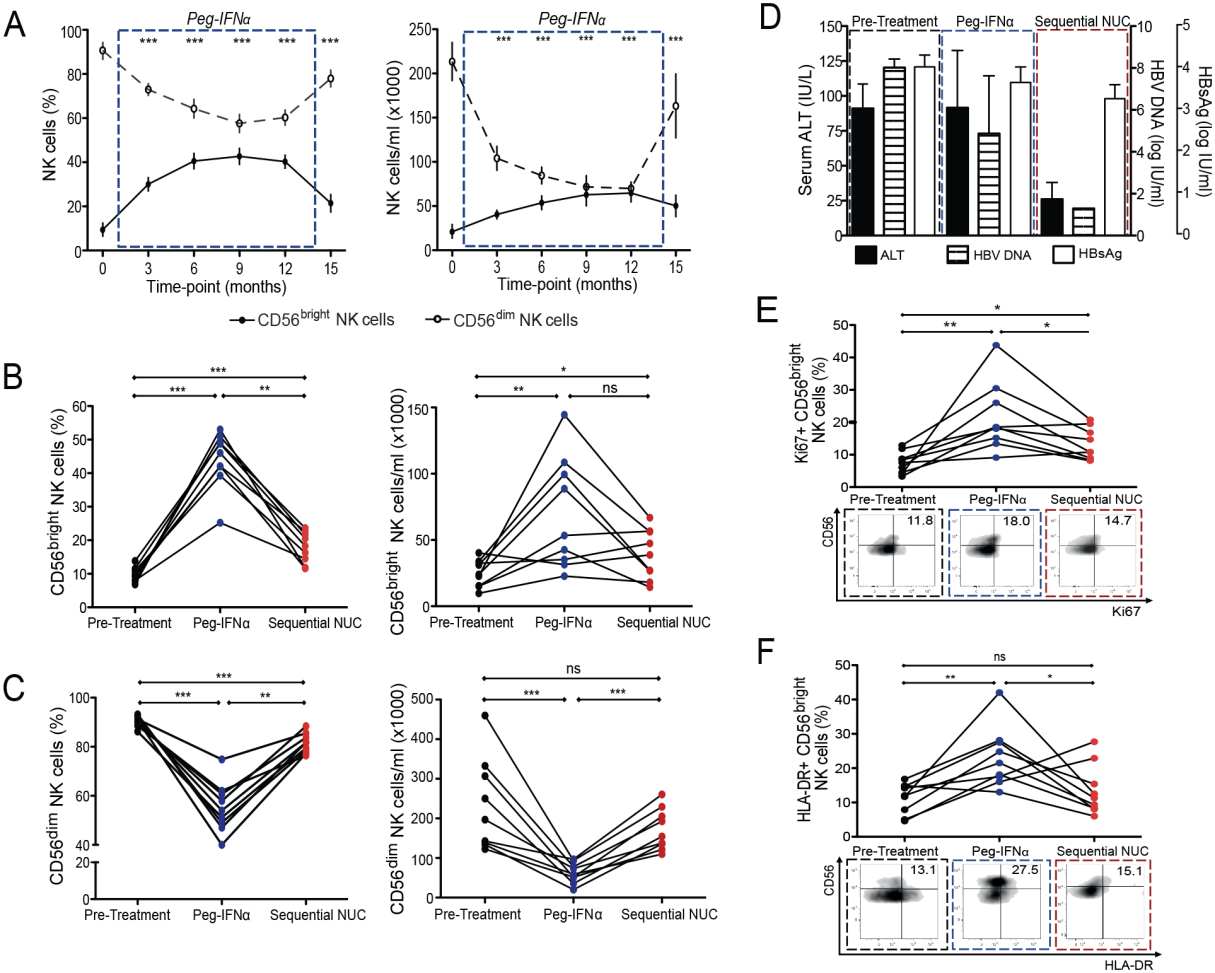
Impact of PegIFNα and sequential NUC therapy on NK cell numbers, proliferation and activation. Cumulative longitudinal data demonstrating change in CD56^bright^ NK cells over the course of PegIFNα therapy by (A) percent and absolute cell number (median ± 95%CI), (n = 18). Change in the number of (B) CD56^bright^ NK cells and (C) CD56^dim^ NK cells (by percent and absolute number) in 9 paired cross-sectional samples in patients on sequential NUC therapy; showing pre-treatment numbers, last sampling treatment time-point on PegIFNα therapy and final sampling time-point on sequential NUC therapy. (D) Corresponding overall ALT, HBV DNA and HBsAg levels (mean + SEM) at the aforementioned sampling time-points. Proportion of CD56^bright^ NK cells expressing (E) Ki67 and (F) HLA-DR, in 9 paired samples, pre-treatment, on PegIFNα and sequential NUC therapy, with representative FACS plots at these time-points. Significant changes marked with asterisks, *P<0.05;**P<0.01; ***P<0.001, ns = not significant.

PegIFNα profoundly expanded NK cells in this cohort of patients with eAg positive CHB, as we had previously reported in eAg negative disease [13]. This expansion was more dramatic for the CD56^bright^ immunoregulatory subset of NK cells, which we have shown to be preferentially enriched in the HBV-infected liver; [12] this subset was therefore studied comprehensively (Figs 2A, 2B and S1A). PegIFNα resulted in a depletion of total circulating lymphocytes; however we confirmed that it induced an increase in both percentage and absolute numbers of CD56^bright^ NK cells (Fig 2A and 2B). The PegIFNα-induced expansion of CD56^bright^ NK cells showed a non-significant trend to decrease on sequential NUCs, but notably, their frequency remained significantly higher than baseline. Conversely the CD56^dim^ subset significantly reduced during PegIFNα therapy and tended to return towards baseline on sequential NUCs (Fig 2C); we therefore focused this study on the CD56^bright^ NK cell subset. ALT normalisation and reduction in HBV DNA was noted, along with a decline in HBsAg levels corresponding to the time-point of viral suppression on sequential NUCs (Fig 2D).

The expansion of CD56^bright^ NK cells on PegIFNα could be attributed to their increased proliferation peaking at 9-months after initiation, as assessed by Ki67 expression, a marker of the replicative S-phase of the cell cycle (Figs 2E and S1B). CD56^bright^ NK proliferation remained significantly higher on sequential NUCs than that observed at baseline, whereas their activation (HLA-DR expression) peaked at 9 months of PegIFNα and was not maintained on sequential NUCs (Fig 2F). By contrast CD56^dim^ NK cells did not have enhanced proliferation or HLA-DR expression during PegIFNα or sequential NUCs (S1D and S1E Fig).

### CD56^bright^ NK cells express high levels of C-type lectin and natural cytotoxicity receptors on sequential NUC therapy

NK cells express a variety of receptors that dictate their activity, [17] hence we analysed their expression of C-type lectin receptors (NKG2A, NKG2C, NKG2D) and natural cytotoxicity receptors (NCRs) (NKp30, NKp44, NKp46).

**Fig 3:**
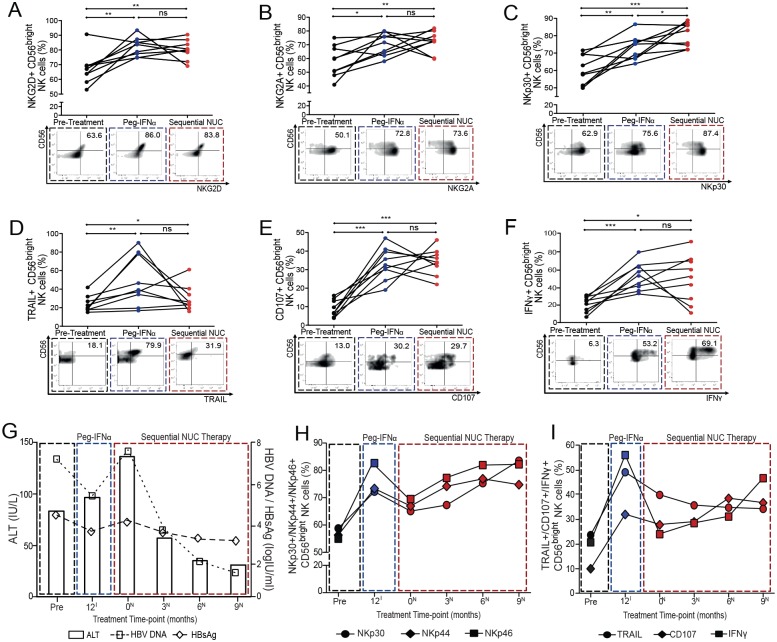
Impact of sequential NUC therapy on the expression of NK cell receptors and functional capacity of NK cells. Percent of CD56^bright^ NK cells expressing (A) NKG2D (B) NKG2A (C) NKp30, (D) TRAIL, (E) CD107 and (F) IFNγ in 9 paired cross-sectional samples pre-treatment, last sampling treatment time-point on PegIFNα therapy and final sampling time-point on sequential NUC therapy with representative FACS plots at these time-points. Significant changes marked with asterisks, *P<0.05;**P<0.01;***P<0.001, ns = not significant. Clinical data showing (G) changes in ALT (IU/L), HBV DNA and HBsAg (log_10_IU/ml) throughout treatments. Corresponding cumulative summary data indicating dynamic changes in (H) NCRs (NKp30, NKp44 and NKp46), (I) TRAIL, CD107 and IFNγ expression throughout treatment, correlating with treatment time-points in (G) (Pre = pre-treatment time-point; 12^I^ = last PegIFNα therapy time-point; 0^N^ = Sequential NUC initiation time-point; 3^N^, 6^N^, 9^N^ = 3, 6 and 9-month sampling time-points on sequential NUC therapy).

We noted an increase in NKG2D and NKG2A on CD56^bright^ NK cells throughout PegIFNα therapy, which remained significantly elevated on sequential NUCs (Figs 3A, 3B, S2A and S2B); no such changes were seen on the CD56^dim^ NK cell subset (S2C and S2D Fig). The expression of the NKG2C receptor was dissimilar. No significant percentage increase in NKG2C was observed during PegIFNα therapy, although an increase in the absolute number of these cells was noted compared to baseline (S2E Fig). Moreover, there was no significant change in the expression of NKG2C during sequential NUCs (S2F and S2G Fig); all patients except for 1 were CMV seropositive so the known influence of CMV on NKG2C could not be discerned in this cohort. NCRs are involved in the clearance of tumour and virus infected cells [18]; in keeping with this, we noted more striking changes in their expression. A significant increase in the expression of NKp30, NKp44 and NKp46 on CD56^bright^ NK cells was seen from 6-months of PegIFNα therapy onwards (Figs 3C and S3A–S3C). Importantly further augmentation of NKp30 expression on CD56^bright^ and CD56^dim^ NK cells was observed on sequential NUCs, peaking at viral suppression, with an inverse temporal relationship noted between its expression and HBV DNA (Figs 3C, 3G, 3H and S3D). Upon sequential NUCs, both NKp44 and NKp46 were maintained on CD56^bright^ NK cells at higher levels than baseline, with expression peaking at 6–9 months, in conjunction with the nadir of HBsAg titre, viral load and ALT (Fig 3G and 3H), but these effects were not seen on the CD56^dim^ NK cells (S3E and S3F Fig).

### NK cell antiviral function is preserved on sequential NUC therapy

The proportion and absolute number of CD56^bright^ NK cells expressing TRAIL increased significantly from 3-months of PegIFNα treatment in eAg positive CHB (in line with previous findings in eAg negative CHB) [13], peaking at 9-months (Figs 3D and S4A), whereas the absolute number of TRAIL+ CD56^dim^ cells did not increase (S4A Fig). TRAIL+ CD56^bright^ NK cells showed a non-significant trend to decrease on sequential NUCs, in line with the reduction of viral load and ALT, but remained significantly higher than pre-treatment levels (Figs 3D and S4D).

PegIFNα induced a potent increase in NK cell degranulation of CD56^bright^ NK cells by percent and absolute number, evident within 3-months of therapy initiation (Figs 3E and S4B); in addition an increase in percent but not absolute number of CD107a+ CD56^dim^ cells was seen (S4B Fig). The ability of CD56^bright^ and CD56^dim^ NK cells to degranulate was maintained on sequential NUCs, peaking at the 6-month time-point (Figs 3E, 3I and S4E).

The cytokine producing CD56^bright^ NK cell subset shows limited ability to produce IFNγ in CHB, [11] however patients with eAg positive CHB achieved a striking recovery of the IFNγ producing capacity of their CD56^bright^ NK cells throughout the course of PegIFNα (Figs 3F and S4C). IFNγ production was maintained on sequential NUCs to a variable degree, at levels significantly higher than baseline (Fig 3F). Improvement in clinical parameters (ALT normalisation, reduction in HBsAg and HBV DNA levels) on sequential NUCs was associated with significantly higher proportion of IFNγ+ CD56^bright^ NK cells at each sequential NUC therapy time-point when compared to NUC initiation (Fig 3F–3H), effects which were not seen on the CD56^dim^ NK cell subset (S4C and S4F Fig).

### Phenotypic and functional capacity of NK cells is greater on sequential NUC therapy compared to de novo NUC therapy or PegIFNα therapy alone

Maintenance of an expanded population of NK cells with altered receptor expression and enhanced function in patients receiving sequential NUCs was noteworthy, contrasting with published findings in patients receiving de novo NUC therapy [11]. To confirm this, we compared the immunological changes documented following sequential NUC therapy (Cohort 1) (S1 Table, Fig 1) with those in virally suppressed patients at a similar time-point on de novo NUC therapy (Cohort 2) (S2 Table, Fig 1). In addition we compared them to patients treated with PegIFNα alone who were sampled 9 months post-treatment (Cohort 3), (S3 Table, Fig 1), to determine if the changes seen on sequential NUCs were merely delayed effects of PegIFNα. Prior to commencing NUC therapy, patients in cohort 1 and 2 had similar HBV DNA levels, and no statistically significant difference in HBsAg concentrations (S1 and S2 Tables).

**Fig 4:**
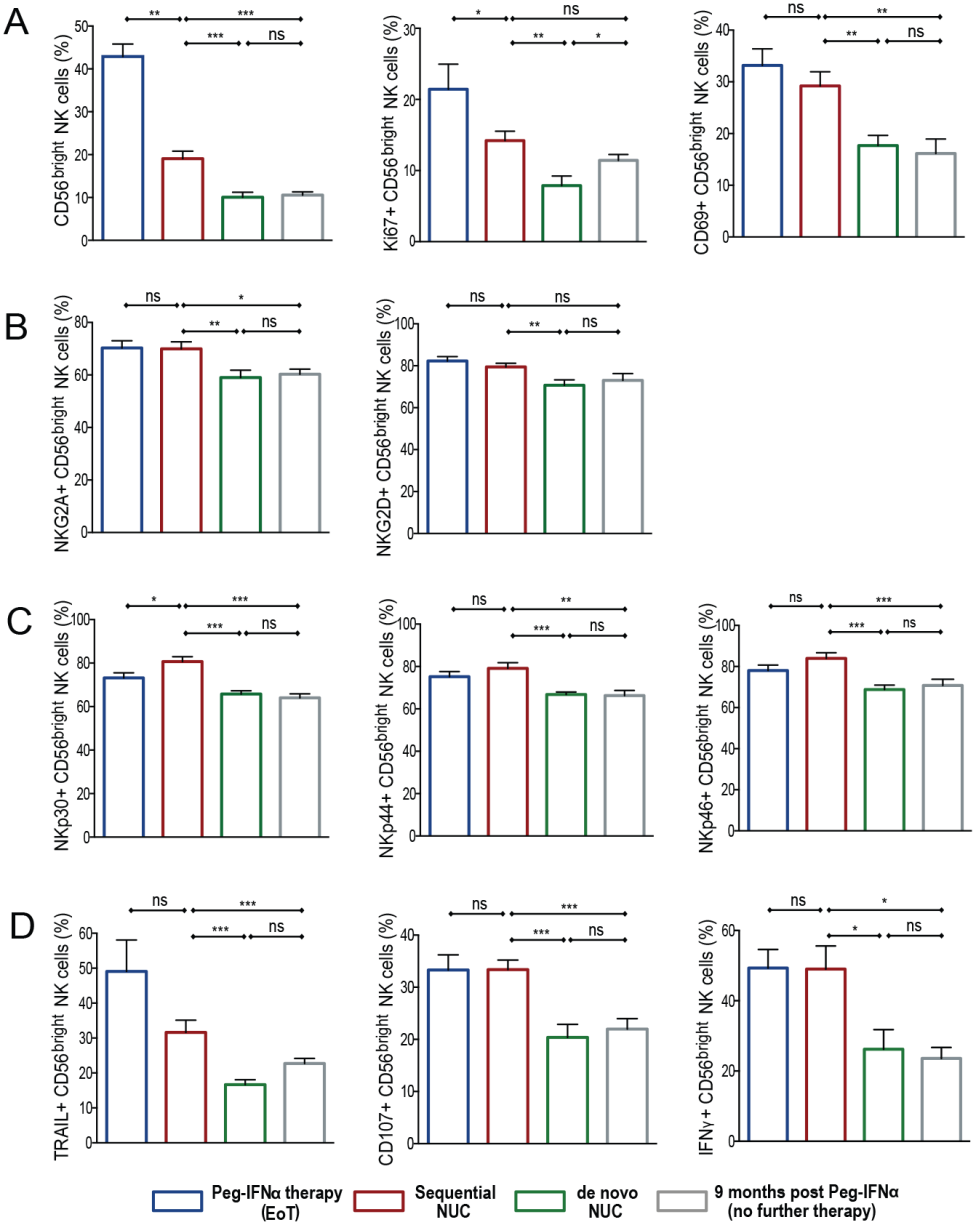
Comparison of NK cells on sequential NUC therapy with de novo NUC therapy & PegIFNα only therapy. Percentage of CD56^bright^ NK cells and markers in the cohort of patients treated with sequential NUC therapy (Cohort 1; n = 14, red outline bars), compared with the cohorts of patients treated with nucleos(t)ide analogues—de novo NUC therapy (Cohort 2; n = 12, green outline bars), without previous PegIFNα exposure, and with PegIFNα alone with no further therapy for 9 months (Cohort 3; n = 10, grey outline bars). Sampling time-point is at viral suppression for patients in cohort 1 and 2. The end of treatment (EoT) PegIFNα sampling time-point for cohort 1, is shown in the blue outline bars for comparison. Markers shown (A) CD56^bright^ NK cells, Ki67, CD69, (B) NKG2A, NKG2D, (C) NKp30, NKp44, NKp46, (D) TRAIL, CD107, IFNγ. Results are expressed as mean ± SEM. Significant changes marked with asterisks, *P<0.05;**P<0.01; ***P<0.001, ns = not significant.

Previous data have shown that CHB patients virally suppressed with de novo NUCs have reduced levels of circulating CD56^bright^ NK cells, similar to those found in healthy individuals [11]. Instead, we noted that patients on sequential NUCs had higher frequencies of CD56^bright^ NK cells, associated with an increase in their proliferative capacity, than those virally suppressed on NUCs alone (Fig 4A). In addition, sequential NUC patients also had a higher frequency of CD56^bright^ NK cells compared with the PegIFNα therapy only cohort (Cohort 3), although there was no significant difference in their proliferation between these cohorts (Fig 4A).

There was no difference in the expression of HLA-DR (S5A Fig), but we did note a higher proportion of CD56^bright^ NK cells expressing the early activation marker, CD69, in patients on sequential NUCs compared to those on de novo NUCs or previous PegIFNα therapy only. In addition the increase in CD69 expression on PegIFNα treatment was maintained on sequential NUCs (Fig 4A). Significantly higher levels of NKG2A, NKG2D and NCR expression were observed in the sequential NUC therapy cohort compared with those on de novo NUCs or the PegIFNα only cohort (Fig 4B and 4C), but no difference was seen in NKG2C expression between the cohorts (S5B Fig). Of particular note, the functional potential of CD56^bright^ NK cells was also increased on sequential NUCs compared to those on de novo NUCs or PegIFNα only. (Fig 4D).

### Evidence of NK cell migration markers seen during sequential NUC therapy

**Fig 5:**
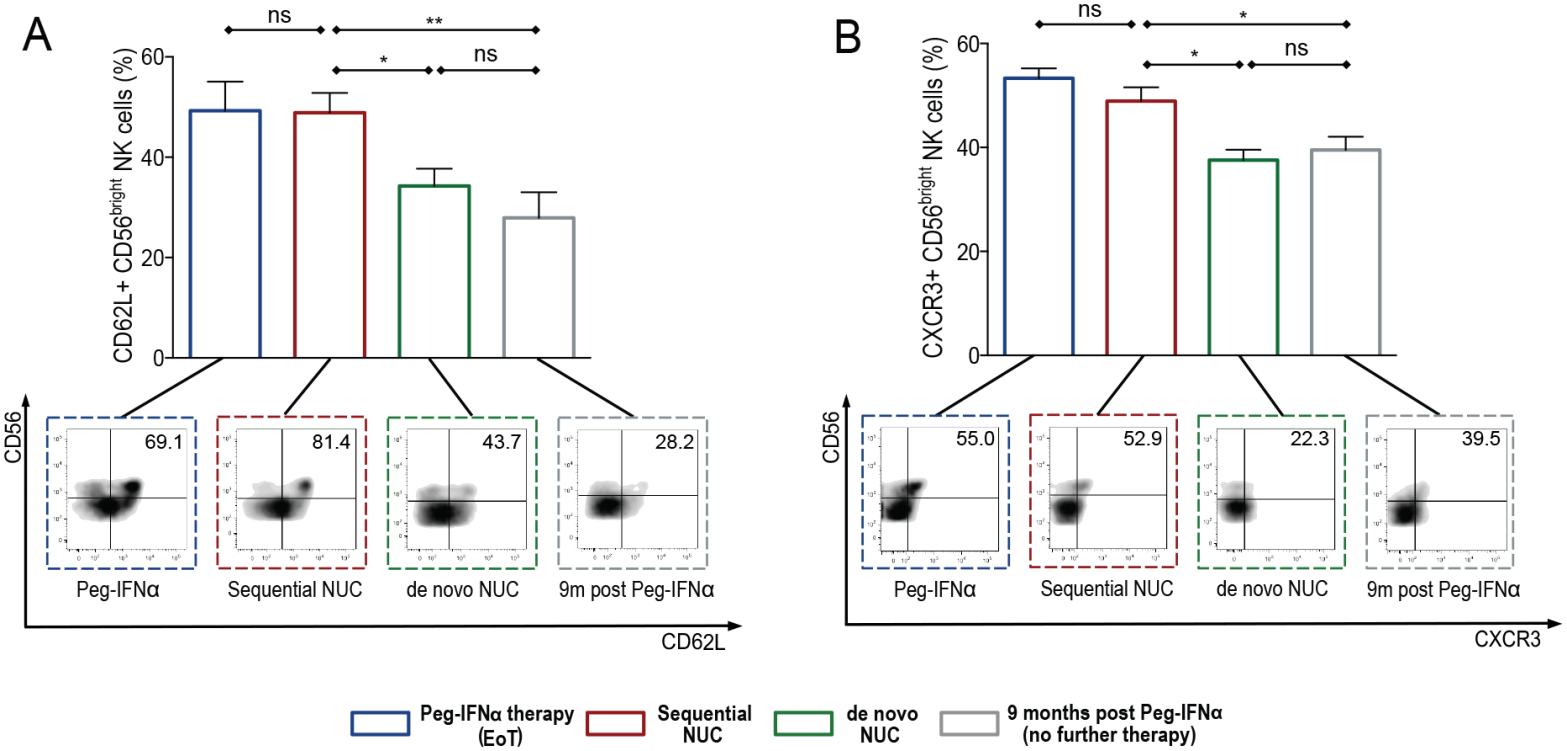
Expression of NK cell migration markers on sequential NUC therapy compared with de novo NUC therapy & PegIFNα only therapy. Percentage of CD56^bright^ NK cells expressing (A) CD62L and (B) CXCR3 in the cohort of patients treated with sequential NUC therapy (Cohort 1; n = 14, red outline bars), compared with the cohorts of patients treated with nucleos(t)ide analogues—de novo NUC therapy (Cohort 2; n = 12, green outline bars), without previous PegIFNα exposure, and with PegIFNα alone with no further therapy for 9 months (Cohort 3; n = 10, grey outline bars). Sampling time-point is at viral suppression for patients in cohort 1 and 2. The end of treatment (EoT) PegIFNα sampling time-point for cohort 1, is shown in the blue outline bars for comparison. Representative FACS plots for each corresponding treatment cohort are shown. Results are expressed as mean ± SEM. Significant changes marked with asterisks, *P<0.05;**P<0.01; ***P<0.001, ns = not significant.

To further characterise NK cells in the therapy cohorts studied, we analysed their expression of tissue homing/migration and maturation markers. CD62L and CCR7 are implicated in NK cell lymph node homing whereas NK cell recruitment to local tissue sites remains less well understood [19]. However chemokine receptors such as CXCR3 [20] and the selectin CD62L [21] have recently been reported to be important for homing and/or protective roles of NK cells in the liver. We found that the expression of CD62L was localised to the CD56^bright^ NK subset and was significantly higher in patients during PegIFNα therapy and upon viral suppression on sequential NUCs, compared with de novo NUCs and after PegIFN alone, (Fig 5A). We did not, however, see similar findings with the expression of CCR7 or CXCR6 on this subset (S5C and S5D Fig). Patients on PegIFNα therapy and sequential NUCs also expressed CXCR3+ CD56^bright^ NK cells at significantly higher levels than those patients virally suppressed on de novo NUCs or after PegIFNα alone (Fig 5B).

The CD56^bright^ NK cells during sequential NUCs were able to express high levels of CD62L, CXCR3 and CD69 (Figs 4A, 5A and 5B), but produced low levels of perforin and granzyme, which localised to the CD56^dim^ NK subset (S5E and S5F Fig). We did not see any change in the proportion of maturation markers on this NK subset, such that there were no differences in CD57 or KLRG1+ CD56^bright/dim^ NK cells in the therapy cohorts, (S5G and S5H Fig) but a modest increase in the CD16+ CD56^bright^ NK cell subsets during PegIFNα and sequential NUCs compared to the other therapy cohorts (S5I Fig).

### Functional NK cell restoration temporally correlates with HBsAg decline on sequential NUC therapy

**Fig 6:**
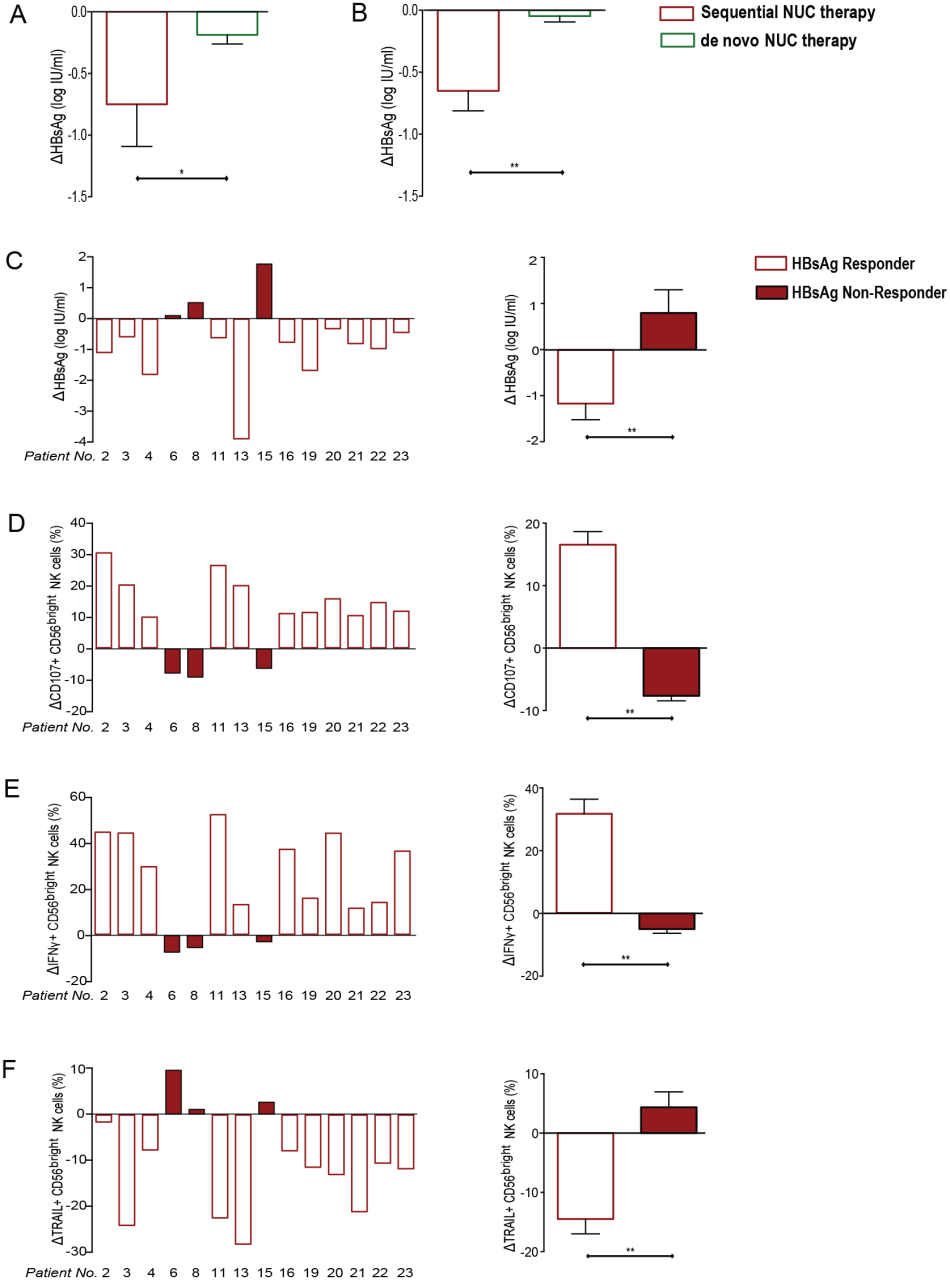
Association of change in HBsAg and functional capacity of NK cells. (A) Change in HBsAg titre (log_10_IU/ml) from initiation of NUC to viral suppression in the sequential NUC therapy cohort (Cohort 1) compared with de novo NUC therapy (Cohort 2). (B) Findings in (A) confirmed in a larger cohort of patients; sequential NUC therapy [n = 28; Baseline (BL): ALT 92 IU/L, HBV DNA 6.65 logIU/ml HBsAg 3.86 logIU/ml], de-novo NUC therapy (n = 30; BL: ALT 70, HBV DNA 5.79 log IU/ml, HBsAg 3.75 logIU/ml); (p = ns for all BL clinical parameters between the cohorts). Results are expressed as mean ± SEM. Change in (C) HBsAg titre (log_10_IU/ml) from initiation of sequential NUC to viral suppression (9-months of sequential NUC therapy in all patients) in the cohort of sequential therapy treated patients (unshaded = HBsAg responder; shaded red = HBsAg non-responder), (Patient number corresponding with number in Table 1 and S1 Table). Change in percentage of (D) CD107+, (E) IFNγ+ and (F) TRAIL+ CD56^bright^ NK cells from initiation of sequential NUC to viral suppression in each patient in the sequential therapy cohort with corresponding summary data for HBsAg responders (unshaded) vs. non-responders (shaded red) for each marker. Significant changes between the groups are marked with asterisks *P<0.05;**P<0.01;***P<0.001, ns = not significant.

Congruent with the enhanced boosting of functional NK cells, patients treated with sequential NUCs achieved a greater decline in HBsAg than those treated for an equivalent duration with de novo NUCs (Fig 6A). To confirm this, we compared the decline in HBsAg achieved after 9–12 months of NUCs in a larger cohort of patients with or without prior PegIFNα; again, the decrease in HBsAg was significantly greater in those on sequential NUCs (Fig 6B).

Marked variability in the maintenance of IFN-induced changes in NK cells in patients on sequential NUCs was noted (Fig 6). We analysed if NK cell functionality was associated with differential clinical outcomes in this small cohort. Patients on sequential NUCs (Cohort 1) were divided according to their HBsAg response; those with any decline in HBsAg from the time of NUC initiation to viral suppression were classified as ‘HBsAg responders’ and those without any decline (or even an increase) in HBsAg as ‘HBsAg non-responders’ (Fig 6C). The overall mean HBsAg decline in the responders (n = 11) was 1.18 logIU/ml, whereas the non-responders (n = 3) demonstrated a 0.80 logIU/ml increase in HBsAg (Fig 6C).

A striking increase in the capacity of CD56^bright^ NK cells to degranulate and produce IFNγ was only seen in those considered HBsAg responders. By contrast HBsAg non-responders showed a significant reduction in NK cell CD107a and IFNγ production, highlighting that functional restoration of NK cells was seen only in HBsAg responders (Fig 6D and 6E). In contrast to other NK cell effector functions, NK cell TRAIL declined in these HBsAg responders on sequential NUCs (Fig 6F). The association of HBsAg decline with a decrease in TRAIL expression points to a possible negative impact of TRAIL on immune reconstitution in the setting of sequential NUCs. This is also in keeping with recent studies, where reduced levels of TRAIL are part of an NK cell phenotype associated with immune control in HBV [15] and sustained virological response following DAA therapy in Hepatitis C virus (HCV) [22,23]. Although the levels of TRAIL remained higher in patients on sequential NUCs following PegIFNα exposure than in the de novo NUC cohort, we noted significant declines in TRAIL+ CD56^bright^ NK cells in the HBsAg responders, with the greatest decline seen in the patient (Pt.13) who lost HBsAg (Fig 6F).

## Discussion

Here we document for the first time that an expanded population of activated, functional NK cells induced by a course of Peg-IFNα can be maintained for at least 9 months after switching to sequential NUCs. This finding is at odds with the traditional view of NK cells as short lived populations with a rapid turnover and contraction following an acute response [24,25] and is instead reminiscent of the prolonged expansion of NK cells reported more recently following homeostatic proliferation in mice [26] and viral infection in humans [27]. Pertinent to this, we have previously shown that PegIFNα treatment of patients with CHB results in sustained induction of IL-15, [13] one of the cytokines pivotal to long-term maintenance of NK cells with ‘recall’ capacity [26,28,29]. In addition a recent study has also demonstrated the role of type-1 IFN in promoting NK cell expansion during viral infections, by protecting them against fratricide [30], which may also be relevant in the setting of sequential NUC therapy.

Our data demonstrate that sustained restoration of NK cell responses is associated with an enhanced decline in HBsAg in a cohort of eAg positive CHB patients exposed to PegIFNα who subsequently progressed to sequential NUC therapy. These data are the first immunological characterisation of effects of this treatment sequence, providing a scientific rationale for further examination of this and other combination or sequential approaches. For example, it maybe possible to achieve similar immunological effects using shorter courses of PegIFNα according to current early stopping rules [7]. Previous studies have focused on the impact of these individual antiviral therapies on the innate [11,13,15] and adaptive [13–15,31] arms of the immune response. Recent data show NUC-induced reductions in viral load could prolong the immunostimulatory effects of PegIFNα given in combination, [32] and that NK cells may play a role in the clearance of HBsAg during these combination therapy strategies [8]. However, no immunological data exist on the impact of PegIFNα ‘priming’ followed by sequential NUC therapy for the management of eAg positive CHB. In this eAg positive CHB cohort, PegIFNα was able to induce a marked expansion of activated CD56^bright^ NK cells with antiviral potential, as we have previously described for patients with eAg negative CHB [13]. Unexpectedly, these profound changes in the NK cell compartment were largely maintained for at least 9 months after switching to sequential NUCs, an effect not seen with de novo NUCs therapy. Thus PegIFNα appeared to ‘prime’ NK cells to sustain long-lasting changes, allowing them to respond differently to NUCs; in patients not exposed to PegIFNα, NK cells demonstrated reduced ability to express activating receptors, tissue homing markers/chemokine receptors, produce IFNγ and degranulate. Interestingly in those patients exposed to PegIFNα only, without further therapy, the functionality of NK cells was not maintained at 9 months post cessation of PegIFNα. This indicates that the effect seen on sequential NUCs is not exclusively related to PegIFNα. Furthermore, the differential effects of NUCs on NK cells based on prior PegIFNα exposure are not explained by differences in baseline viral load or HBsAg levels in these cohorts. However, sequential NUCs were associated with significantly greater declines in HBsAg, although no correlation was noted with eAg seroconversion, when compared to de novo NUCs, which may have been attributable to the NK cell reconstitution and/or have contributed to it. These changes merit further study in a larger cohort.

It is noteworthy nonetheless, that within this small cohort, reductions in HBsAg were temporally associated with increases in NK cell cytotoxicity and IFNγ production, but with reductions in TRAIL expression, suggesting that the latter may be pathogenic in the setting of sequential NUCs. We have recently reported that TRAIL-bearing NK cells can delete HBV-specific T cells and could therefore constrain antiviral T cell immunity [33]. In keeping with these data, a recent study has shown that NK cell-TRAIL blockade may also lead to recovery of HBV-specific T cells in eAg negative patients virally suppressed on NUCs [15]. Similarly, reduced levels of NK cell TRAIL are associated with sustained virological response following DAA therapy in HCV [22,23]. Although we did not see TRAIL levels return to baseline in the sequential therapy cohort, we note that there were significant declines in TRAIL+ CD56^bright^ NK cells in the HBsAg responders, with the greatest decline seen in the one patient who lost HBsAg.

Not only were IFNα-induced changes in NK cells maintained on sequential NUCs, but the expression of the activating NCRs, NKp30 and NKp46 were further enhanced on sequential NUCs, showing an inverse temporal correlation with HBV DNA. Recent data from treated cohorts in hepatitis C and delta virus have also demonstrated the modulation of these receptors [22,34–38]. NKp30 has been shown to be pivotal in NK/dendritic cell cross-talk [39,40] and the regulation of NK cell IFNγ production; [41] consistent with this, we found that enhanced IFNγ production reflected NKp30 expression on sequential NUCs. The correlation we observed between increases in NK cell NKp46 and decline in HBsAg is also in line with data from the HCV field linking this activating receptor with cytotoxic, [42] antiviral and anti-fibrotic activity of NK cells [43–45]. In keeping with this, the increased expression of CXCR3+ CD56^bright^ NKs, seen on sequential NUCs, may be implicated in anti-fibrogenesis [20] in HBV therapies, along with the increased expression of the tissue homing markers, CD62L and CD69, which may be involved with hepatic NK cell recruitment. Further studies of the ‘on-treatment’ liver compartment would be of interest to fully elucidate this role.

Despite the limited number of patients studied, these novel data highlight the potential immunological benefits of PegIFNα-priming as part of a therapeutic strategy. Recent data from the woodchuck hepatitis model show the induction of a T/NK cell signature in the liver correlating with treatment outcome [46]. This highlights the potential therapeutic role for PegIFNα-priming and the resulting modulation of the immune response. Combination or sequential therapy regimes with PegIFNα and NUCs have been postulated to have the capacity to exert complementary or synergistic antiviral and immunomodulatory effects. Previous studies of combination therapies have shown promise in reducing the amount of HBcAg+ hepatocytes and cccDNA loads, [47,48] harnessing the different antiviral mechanisms of PegIFNα and NUCs [7,8,49,50]. Such regimes should likewise take advantage of the ability of PegIFNα [51] and NUCs [14,15] to reconstitute the innate and adaptive arms of the immune response respectively [7,13–15]. Our analysis of the virus specific T-cell response in these patient cohorts, albeit limited, has demonstrated their very low frequency during PegIFNα, with significant recovery on sequential NUCs (S6 Fig). However, future studies will be required to determine whether the capacity of NUCs to induce T cell reconstitution is altered by PegIFNα-priming and its potential effects on NK and T cell interactions [33]. In summary our study supports the capacity of human NK cells to undergo long-lived changes in the context of in vivo IFNα exposure followed by NUC therapy and provides a mechanistic rationale for sequential therapy with PegIFNα followed by NUCs.

## Materials and Methods

### Ethics statement

Clinical assessment and additional blood sampling were performed during routine hepatitis/treatment clinics at The Royal London Hospital. Written informed consent was obtained and the study was approved by the local ethics committee (Barts and The London NHS Trust Ethics Review Board).

### Patient samples/study design

Forty-five eAg positive CHB patients undergoing standard HBV treatment regimes were recruited for immunological analysis. Baseline HBV serology was measured, including HBV DNA levels, quantified by real-time PCR (Roche COBAS AmpliPrep/COBAS Taqman HBV test v2.0-dynamic range 20 to 1.7x10^8^ IU/ml-Roche molecular diagnostics, Pleasanton, CA) and HBsAg titre (Abbott Architect). Serum was also tested for HBeAg and anti-HBe with a chemiluminescent microparticle immunoassay (Abbott Architect, Abbott Diagnostics, Abbot Park, IL) and CMV IgG. HBV genotype was recorded, along with serum transaminases and Ishak fibrosis stage where liver biopsies were performed.

18 consecutive patients were treated with a 48-week course of Pegylated Interferon-α 2a (180μg/week-Pegasys) as first-line therapy (Cohort 1). Treatment responses were defined in accordance with national and international guidelines [2–5] and those considered PegIFNα failures/non-responders 6–12 weeks following cessation of PegIFNα (determined by viral rebound, ALT flare or both), (14/18 patients) were offered Entecavir or Tenofovir; defined as sequential NUC therapy for the purpose of this study (taken up by nine of these patients). Detailed longitudinal sampling was carried out on patients from Cohort 1 to characterise temporal immunological changes throughout PegIFNα and sequential NUC therapy. In addition a further 5 patients, deemed treatment failures, following a 48-week course of PegIFNα, progressed to sequential NUC therapy, and were also studied, prior to starting NUC and at viral suppression. (Fig 1, Table 1 and S1 Table). Immune changes after 9 months of sequential NUCs, in this cohort (n = 14), were compared cross-sectionally with the following control cohorts sampled at an equivalent time point: Control Cohort 2; 12 patients treated with de novo NUC therapy (Fig 1, S2 Table). Control Cohort 3; 10 patients treated with PegIFNα for 48 weeks, (responders, n = 4; non-responders, n = 6), sampled 9 months after cessation of PegIFNα (Fig 1, S3 Table).

### Extracellular staining of NK and T cells

For phenotypic analysis of NK cells, PBMC were stained with the following fluorochrome conjugated antibodies or isotype matched controls: CD3-Cy5.5/PerCP or CD3/Pe-Cy7, (eBioscience, Hatfield, UK), CD56-PE Texas Red (Beckman Coulter, High Wycombe, UK), CD56-FITC, CD16-APC Cy7, HLA-DR V500, CXCR3-Cy.5./PerCP, CD57-BV605, NKp46-V450, TRAIL-PE, (BD Biosciences, Oxford, UK), TRAIL-BV421, CD62L-AF700, CXCR6-PE, CD69-APC, CCR7-BV421, KLRG1-FITC, (Biolegend, London, UK), NKG2A-APC, NKG2C-Cy5.5/PerCP, NKG2D-PE (R&D systems, Abingdon, UK), NKp30-APC, NKp44-PE (Miltenyi Biotec, Surrey, UK), in the presence of fixable live/dead stain (Invitrogen, Paisley, Scotland). For phenotypic analysis of T cells PBMC were stained with the fluorochrome conjugated antibodies or isotype matched controls: CD3/Pe-Cy7, CD8-AF700, CD4-APC Cy7 (eBioscience, Hatfield, UK), CD38-PE Texas Red, CD14-V500, CD19-V500 (Biolegend, London, UK). Flourescence minus-one (FMOs) were used for gating purposes for all flourochromes; an example of the gating strategy is shown in S1A Fig. Cells were acquired on a FACS LSRII multicolour flow cytometer (Beckton Dickinson) and analysed using Flow Jo analysis software (Tree star, Ashland, OR, USA). In addition to percentage, absolute numbers of NK cell subsets were calculated by multiplying their percent by total lymphocyte count.

### Intracellular staining and effector functions of NK cells

To assess proliferation and further characterisation of the differentiation of NK cells, PBMC were permeabilised and stained with anti-Ki67-PE (eBioscience, Hatfield, UK), Granzyme-B-FITC and Perforin-Cy5.5/PerCP (Biolegend, London, UK) directly ex-vivo. For intracellular staining for IFNγ production; PBMC were incubated with rhIL12 and rhIL15 (10ng/ml) (R&D systems, Abingdon, UK), for 19 hours at 37°C. 1mM monensin (Sigma-Aldrich, Gillingham, UK) was added for the final 3 hours. Cells were then stained with anti-CD3-Cy5.5/PerCP or CD3/Pe-Cy7, CD16-APCy7, CD56-FITC, and subsequently fixed and permeabilised, followed by intracellular staining for IFNγ-v450 (BD Biosciences, Oxford, UK). Dead cells were excluded by fixable live dead stain.

For degranulation, PBMCs were incubated with K562 cells (5:1 E:T ratio) for 3 hours following overnight stimulation with a combination of 50ng/ml rhIL12 and rhIL18 (Miltenyi Biotech). Anti-CD107a-PE mAb (BD Biociences, Oxford, UK) was added at the time of stimulation with target-cells and monensin (1mM) added during the last 2 hours of incubation prior to staining and acquisition.

### Virus-specific CD8 T cell analysis

Patients were tested for their HLA-A2 status and PBMC from HLA-A2+ patients were stimulated with peptides representing HLA-A2-restricted HBV epitopes (HBVenv: FLLTRILTI, WLSLLVPFV, LLVPFVQWFV, GLSPTVWLSV; HBVcore: FLPSDFFPSV; HBVpol: GLSRYVARL, KLHLYSHPI) or the CMV pp65-encoded NLVPMVATV epitope (Proimmune). Virus-specific cells were identified by multicolour flow-cytometry (BD LSR II): surface staining with CD3/Pe-Cy7, CD8-AF700, CD4-APC Cy7 (eBioscience, Hatfield, UK), CD38-PE Texas Red, CD14-V500, CD19-V500 (Biolegend, London, UK) in the presence of fixable live/dead stain (Invitrogen).

### Statistical analysis

Significance was performed between paired samples; (pre-treatment, on PegIFNα therapy and on sequential therapy), in addition to longitudinal analysis of samples using repeated Anova measurements. *P*<0.05 was considered significant in all cases. (Prism version 5, GraphPad Software Inc., San Diego, Calif.).

## Supporting Information

S1 Fig Impact of PegIFNα therapy and sequential NUC therapy on proliferative capacity and activation of NK cells. (A) Gating strategy for identification of NK cells and markers (singlets, total lymphocytes, live cells, CD3- CD56+ cells) using multicolour flow cytometry, gating with FMO and mAb shown for CD62L as an example; all other markers analysed using the same gating strategy. Cumulative longitudinal data demonstrating change in (B) Ki67+ and (C) HLA-DR+ CD56^bright^ and CD56^dim^ NK cells over the course of PegIFNα therapy by percent and absolute cell number (median ± 95%CI), (n = 18). Percent of (D) Ki67+ and (E) HLA-DR+ CD56^dim^ NK cells pre-treatment, the last sampling time-point of PegIFNα and at viral suppression on sequential NUC therapy (significant increases marked with asterisks; *P<0.05;**P<0.01;***P<0.001, ns = not significant).
(PDF)

S2 Fig Impact of PegIFNα therapy and sequential NUC therapy on C-type lectin receptor expression. Cumulative longitudinal data demonstrating change in (A) NKG2D+ and (B) NKG2A+ CD56^bright^ and CD56^dim^ NK cells over the course of PegIFNα therapy by percent and absolute cell number (median ± 95%CI), (n = 18). Percent of (C) NKG2D+ and (D) NKG2A+ CD56^dim^ NK cells pre-treatment, the last sampling time-point of PegIFNα and at viral suppression on sequential NUC therapy. Cumulative longitudinal data demonstrating change in (E) NKG2C+ CD56^bright^ and CD56^dim^ NK cells over the course of PegIFNα therapy by percent and absolute cell number (median ± 95%CI), (n = 18). Percent of (F) NKG2C+ CD56^bright^ and (G) NKG2C+ CD56^dim^ NK cells in 9 paired cross-sectional samples pre-treatment, the last sampling time-point of PegIFNα and at viral suppression on sequential NUC therapy with representative FACS plots at these time-points. (Significant increases marked with asterisks; *P<0.05;**P<0.01;***P<0.001, ns = not significant).
(PDF)

S3 Fig Impact of PegIFNα therapy and sequential NUC therapy on NCR expression. Cumulative longitudinal data demonstrating change in (A) NKp30+, (B) NKp44+ and (C) NKp46+ CD56^bright^ and CD56^dim^ NK cells over the course of PegIFNα therapy by percent and absolute cell number (median ± 95%CI), (n = 18). Percent of (D) NKp30+, (E) NKp44+ and (F) NKp46+ CD56^dim^ NK cells pre-treatment, the last sampling time-point of PegIFNα and at viral suppression on sequential NUC therapy (significant increases above baseline marked with asterisks; *P<0.05; **P<0.01;***P < .001, ns = not significant).
(PDF)

S4 Fig Impact of PegIFNα therapy and sequential NUC therapy on the functional capacity of NK cells. Cumulative longitudinal data demonstrating change in (A) TRAIL+, (B) CD107+ and (C) IFNγ+ CD56^bright^ and CD56^dim^ NK cells over the course of PegIFNα therapy by percent and absolute cell number (median ± 95%CI), (n = 18). Percent of (D) TRAIL+, (E) CD107+ and (F) IFNγ+ CD56^dim^ NK cells pre-treatment, the last sampling time-point of PegIFNα and at viral suppression on sequential NUC therapy (significant increases above baseline marked with asterisks; *P<0.05;**P<0.01;***P<0.001, ns = not significant).
(PDF)

S5 Fig Comparison of markers of activation, migration, cytotoxicity and maturation during sequential NUC therapy compared with de novo NUC therapy and PegIFNα only therapy. Percentage of: (A) HLA-DR+, (B) NKG2C+ CD56^bright^ NK cells, markers of migration; C) CCR7+ and (D) CXCR6+ CD56^bright^ and CD56^dim^ NK cells, (E) Perforin+ and (F) Granzyme+ CD56^bright^ and CD56^dim^ NK cells and markers of maturation; (G) CD57+, (H) KLRG1+ and (I) CD16+ CD56^bright^ and CD56^dim^ NK cells from patients in each treatment cohort (as in Fig 1). Sequential NUC therapy (Cohort 1; n = 14, red outline bars), compared with the cohorts of patients treated with nucleos(t)ide analogues—de novo NUC therapy (Cohort 2; n = 12, green outline bars), without previous PegIFNα exposure, and with PegIFNα alone with no further therapy for 9 months (Cohort 3; n = 10, grey outline bars). Sampling time-point is at viral suppression for patients in cohort 1 and 2. The end of treatment (EoT) PegIFNα sampling time-point for cohort 1, is shown in the blue outline bars for comparison. Results are expressed as mean ± SEM. Significant changes marked with asterisks, *P<0.05;**P<0.01; ***P<0.001, ns = not significant.
(PDF)

S6 Fig Impact of differing therapies on T cell numbers. Percentage of (A) CD8+ and (B) CD4+ T cells. Patients from each cohort were tested for HLA-A2 status; positive patients (see Supporting Tables) were tested for HBV-specific T cells, (C) Representative FACS plots and summary data of HBV-specific CD8+ T cells, in the cohort of patients treated with sequential NUC therapy (Cohort 1; n = 14, HLA-A2+; n = 5, red outline bars), compared with the cohorts of patients treated with nucleos(t)ide analogues—de novo NUC therapy (Cohort 2; n = 12, HLA-A2+; n = 5, green outline bars), without previous PegIFNα exposure, and with PegIFNα alone with no further therapy for 9 months (Cohort 3; n = 10, HLA-A2+; n = 4, grey outline bars). Sampling time-point is at viral suppression for patients in cohort 1 and 2. The end of treatment (EoT) PegIFNα sampling time-point for cohort 1 is shown in the blue outline bars for comparison (n = 14, HLA-A2+ n = 5). Results are expressed as mean ± SEM. Significant changes marked with asterisks, *P<0.05;**P<0.01; ***P<0.001, ns = not significant.
(PDF)

S1 Table Clinical parameters of sequential NUC therapy patients (Cohort 1). Numbers in brackets under headings; ALT, HBV DNA & HBsAg are mean values. * denotes ALT, HBV DNA & HBsAg at time of sequential NUC initiation. ** denotes ALT, HBV DNA & HBsAg at time of viral suppression. ^ denotes sustained HBsAg loss and ^§^ are patients sampled only at selected time-points on sequential NUC therapy.
(PDF)

S2 Table Clinical parameters of de novo NUC therapy patients (Cohort 2). Numbers in brackets under headings; age is the median value for the cohort, ALT, HBV DNA & HBsAg are mean values. * denotes ALT, HBV DNA & HBsAg at time of de novo NUC initiation. ** denotes ALT, HBV DNA & HBsAg at time of viral suppression. ND–Test not done.
(PDF)

S3 Table Clinical parameters of Peg-IFNα only therapy patients (Cohort 3). Numbers in brackets under headings; age is the median value for the cohort, ALT, HBV DNA & HBsAg are mean values. * denotes ALT, HBV DNA & HBsAg at baseline; prior to Peg-IFNα initiation. ** denotes ALT, HBV DNA & HBsAg at sampling time point (9–12 months post cessation of Peg-IFNα). # denotes sustained HBeAg seroconversion & HBV DNA <2000 IU/ml 6-months post cessation of Peg-IFNα, † indicates patients that refused sequential NUC therapy.
(PDF)
