# Supplementary figures and images for: Interferon Alpha Induces Sustained Changes in NK Cell Responsiveness to Hepatitis B Viral Load Suppression In Vivo
Source: PLoS Pathog. 2016 Aug 3;12(8):e1005788. doi: 10.1371/journal.ppat.1005788 (PMC4972354; doi:10.1371/journal.ppat.1005788)

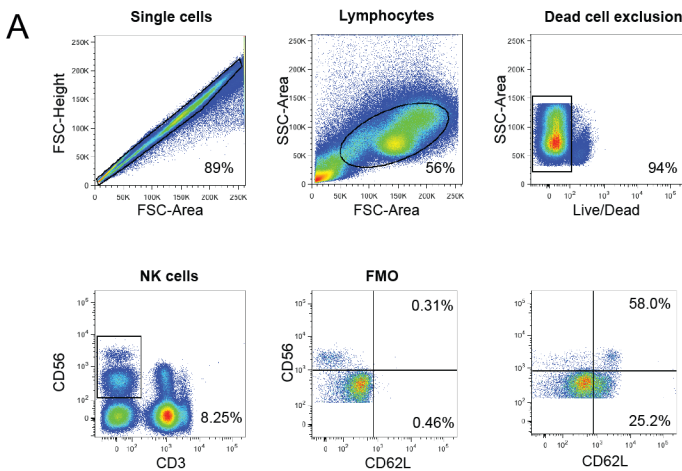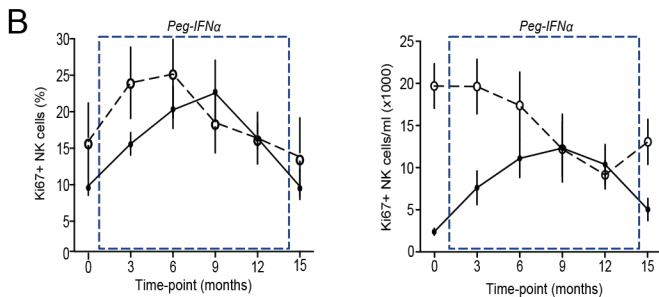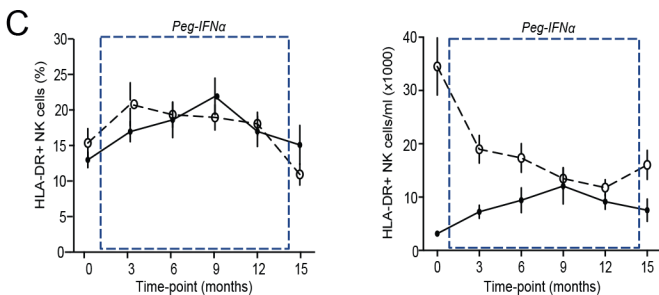

—●— CD56<sup>bright</sup> NK cells  
 —○— CD56<sup>dim</sup> NK cells

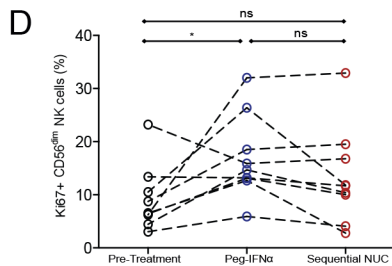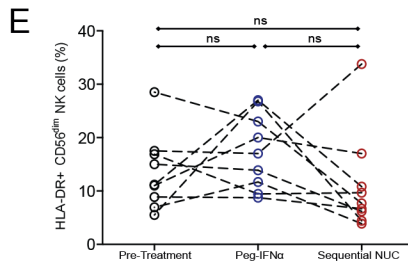

Supplement: S1 Fig — (A) Gating strategy for identification of NK cells and markers (singlets, total lymphocytes, live cells, CD3- CD56+ cells) using multicolour flow cytometry, gating with FMO and mAb shown for CD62L as an example; all other markers analysed using the same gating strategy. Cumulative longitudinal data demonstrating change in (B) Ki67+ and (C) HLA-DR+ CD56bright and CD56dim NK cells over the course of PegIFNα therapy by percent and absolute cell number (median ± 95%CI), (n = 18). Percent of (D) Ki67+ and (E) HLA-DR+ CD56dim NK cells pre-treatment, the last sampling time-point of PegIFNα and at viral suppression on sequential NUC therapy (significant increases marked with asterisks; *P<0.05;**P<0.01;***P<0.001, ns = not significant). (PDF) [file ppat.1005788.s001.pdf]

**A**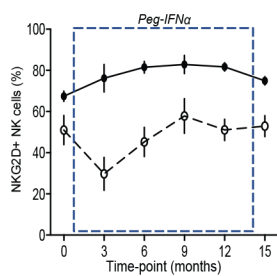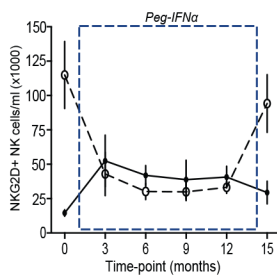**C**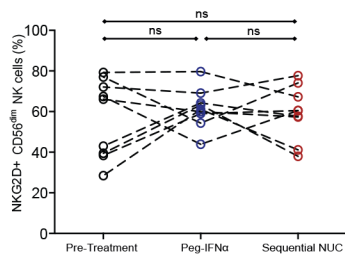**B**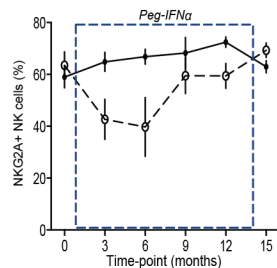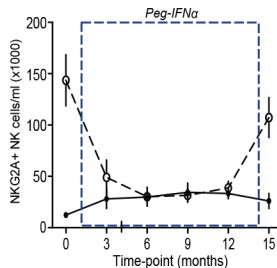**D**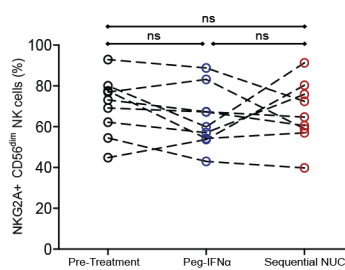**E**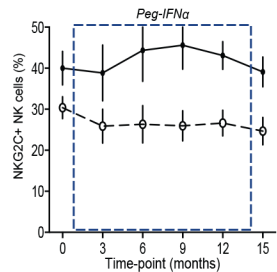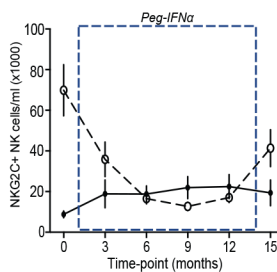

CD56<sup>bright</sup> NK cells

CD56<sup>dim</sup> NK cells

**F**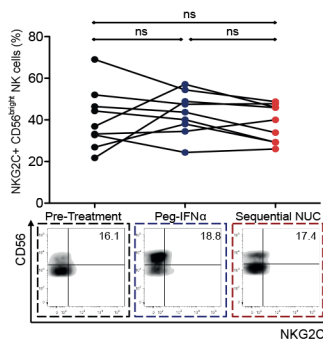**G**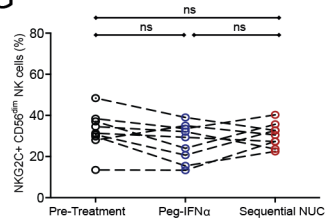

Supplement: S2 Fig — Cumulative longitudinal data demonstrating change in (A) NKG2D+ and (B) NKG2A+ CD56bright and CD56dim NK cells over the course of PegIFNα therapy by percent and absolute cell number (median ± 95%CI), (n = 18). Percent of (C) NKG2D+ and (D) NKG2A+ CD56dim NK cells pre-treatment, the last sampling time-point of PegIFNα and at viral suppression on sequential NUC therapy. Cumulative longitudinal data demonstrating change in (E) NKG2C+ CD56bright and CD56dim NK cells over the course of PegIFNα therapy by percent and absolute cell number (median ± 95%CI), (n = 18). Percent of (F) NKG2C+ CD56bright and (G) NKG2C+ CD56dim NK cells in 9 paired cross-sectional samples pre-treatment, the last sampling time-point of PegIFNα and at viral suppression on sequential NUC therapy with representative FACS plots at these time-points. (Significant increases marked with asterisks; *P<0.05;**P<0.01;***P<0.001, ns = not significant). (PDF) [file ppat.1005788.s002.pdf]

A

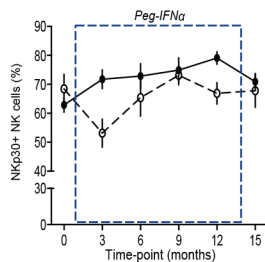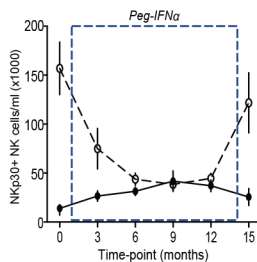

D

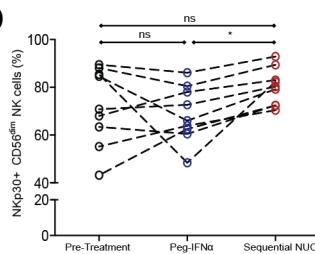

B

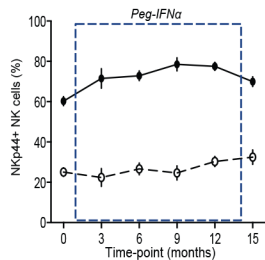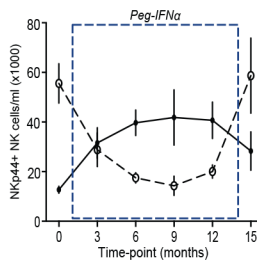

E

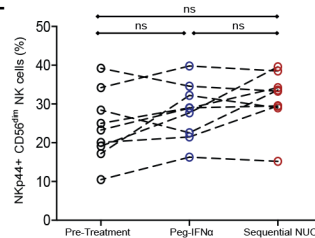

C

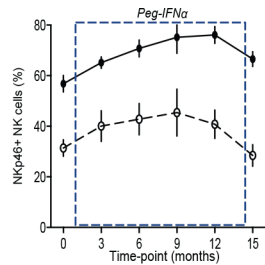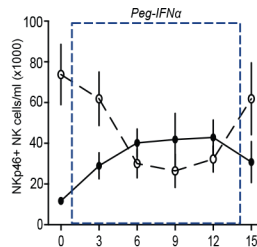

F

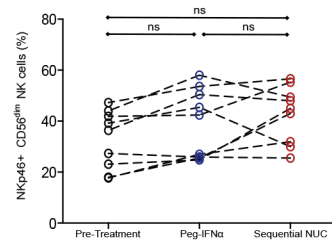

—●— CD56<sup>high</sup> NK cells

—○— CD56<sup>dim</sup> NK cells

Supplement: S3 Fig — Cumulative longitudinal data demonstrating change in (A) NKp30+, (B) NKp44+ and (C) NKp46+ CD56bright and CD56dim NK cells over the course of PegIFNα therapy by percent and absolute cell number (median ± 95%CI), (n = 18). Percent of (D) NKp30+, (E) NKp44+ and (F) NKp46+ CD56dim NK cells pre-treatment, the last sampling time-point of PegIFNα and at viral suppression on sequential NUC therapy (significant increases above baseline marked with asterisks; *P<0.05; **P<0.01;***P < .001, ns = not significant). (PDF) [file ppat.1005788.s003.pdf]

**A**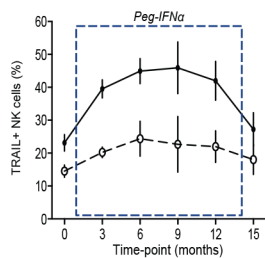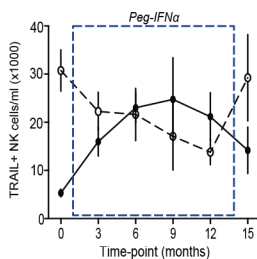**B**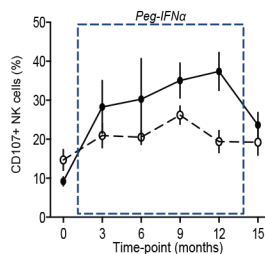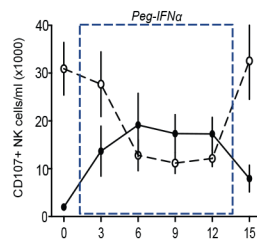**C**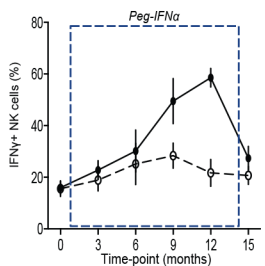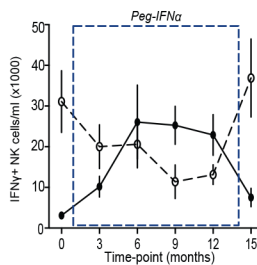

—●— CD56<sup>high</sup> NK cells  
 -○- CD56<sup>dim</sup> NK cells

**D**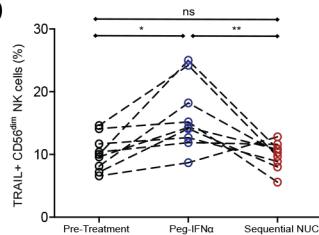**E**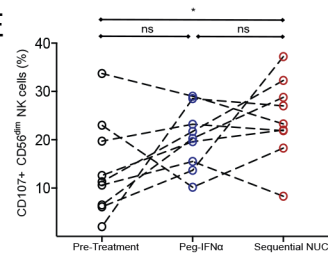**F**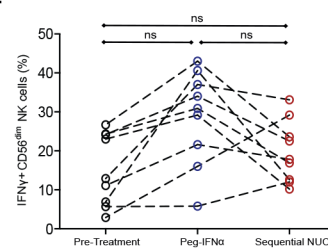

Supplement: S4 Fig — Cumulative longitudinal data demonstrating change in (A) TRAIL+, (B) CD107+ and (C) IFNγ+ CD56bright and CD56dim NK cells over the course of PegIFNα therapy by percent and absolute cell number (median ± 95%CI), (n = 18). Percent of (D) TRAIL+, (E) CD107+ and (F) IFNγ+ CD56dim NK cells pre-treatment, the last sampling time-point of PegIFNα and at viral suppression on sequential NUC therapy (significant increases above baseline marked with asterisks; *P<0.05;**P<0.01;***P<0.001, ns = not significant). (PDF) [file ppat.1005788.s004.pdf]

A

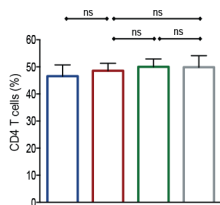

B

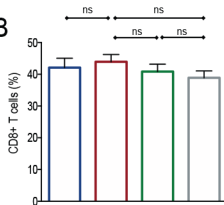

C

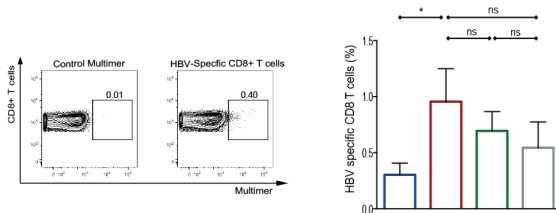

█ Peg-IFNα therapy (EoT)
 █ Sequential NUC
 █ de novo NUC
 █ 9 months post Peg-IFNα (no further therapy)

Supplement: S6 Fig — Percentage of (A) CD8+ and (B) CD4+ T cells. Patients from each cohort were tested for HLA-A2 status; positive patients (see Supporting Tables) were tested for HBV-specific T cells, (C) Representative FACS plots and summary data of HBV-specific CD8+ T cells, in the cohort of patients treated with sequential NUC therapy (Cohort 1; n = 14, HLA-A2+; n = 5, red outline bars), compared with the cohorts of patients treated with nucleos(t)ide analogues—de novo NUC therapy (Cohort 2; n = 12, HLA-A2+; n = 5, green outline bars), without previous PegIFNα exposure, and with PegIFNα alone with no further therapy for 9 months (Cohort 3; n = 10, HLA-A2+; n = 4, grey outline bars). Sampling time-point is at viral suppression for patients in cohort 1 and 2. The end of treatment (EoT) PegIFNα sampling time-point for cohort 1 is shown in the blue outline bars for comparison (n = 14, HLA-A2+ n = 5). Results are expressed as mean ± SEM. Significant changes marked with asterisks, *P<0.05;**P<0.01; ***P<0.001, ns = not significant. (PDF) [file ppat.1005788.s006.pdf]
