# Supplementary material for: Interferon Alpha Induces Sustained Changes in NK Cell Responsiveness to Hepatitis B Viral Load Suppression In Vivo
Source: PLoS Pathog. 2016 Aug 3;12(8):e1005788. doi: 10.1371/journal.ppat.1005788 (PMC4972354; doi:10.1371/journal.ppat.1005788)
Supplement: S5 Fig — Percentage of: (A) HLA-DR+, (B) NKG2C+ CD56bright NK cells, markers of migration; C) CCR7+ and (D) CXCR6+ CD56bright and CD56dim NK cells, (E) Perforin+ and (F) Granzyme+ CD56bright and CD56dim NK cells and markers of maturation; (G) CD57+, (H) KLRG1+ and (I) CD16+ CD56bright and CD56dim NK cells from patients in each treatment cohort (as in Fig 1). Sequential NUC therapy (Cohort 1; n = 14, red outline bars), compared with the cohorts of patients treated with nucleos(t)ide analogues—de novo NUC therapy (Cohort 2; n = 12, green outline bars), without previous PegIFNα exposure, and with PegIFNα alone with no further therapy for 9 months (Cohort 3; n = 10, grey outline bars). Sampling time-point is at viral suppression for patients in cohort 1 and 2. The end of treatment (EoT) PegIFNα sampling time-point for cohort 1, is shown in the blue outline bars for comparison. Results are expressed as mean ± SEM. Significant changes marked with asterisks, *P<0.05;**P<0.01; ***P<0.001, ns = not significant. (PDF) [file ppat.1005788.s005.pdf]

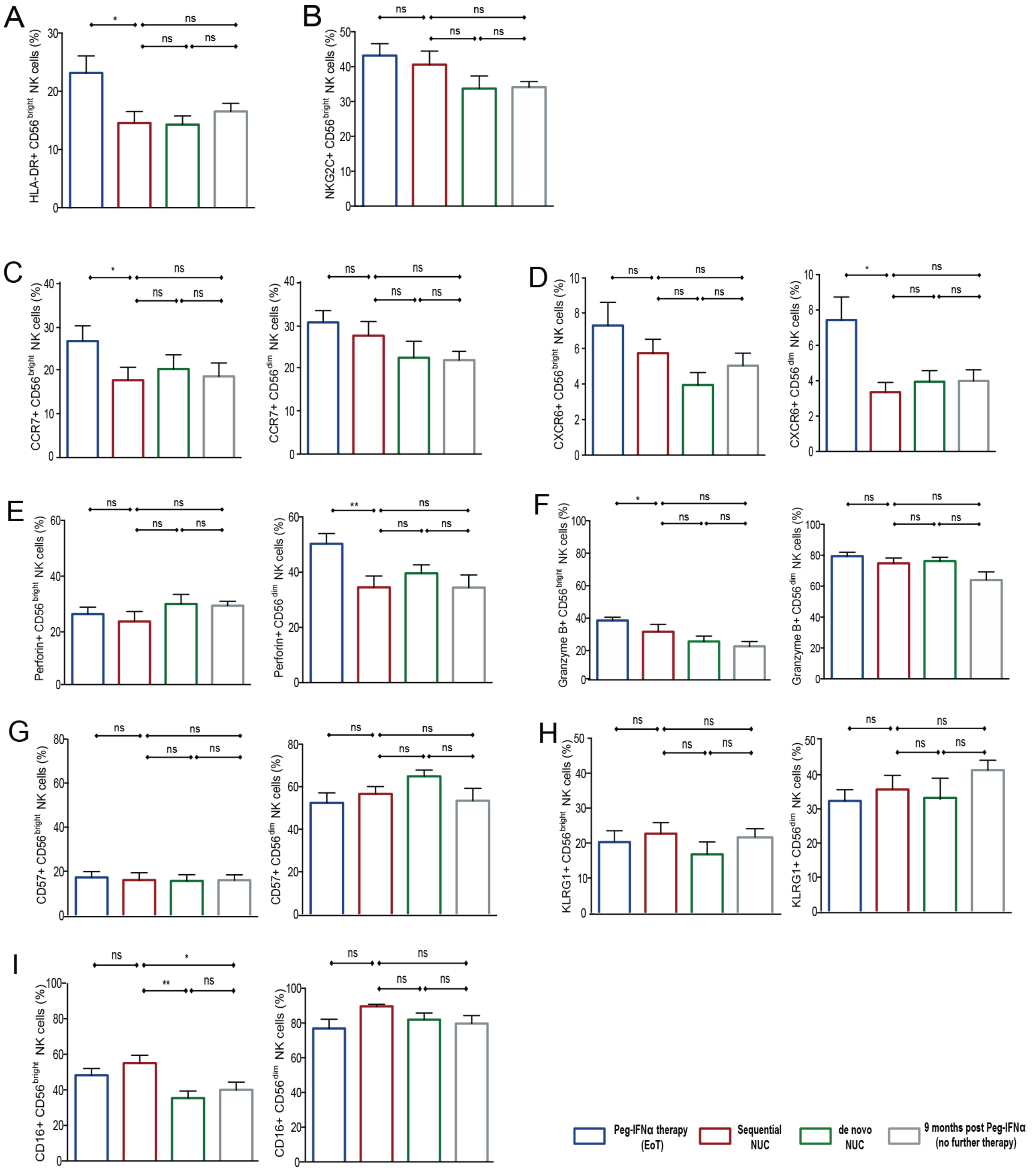

A
HLA-DR+ CD56bright NK cells (%)
B
NKG2C+ CD56bright NK cells (%)
C
CCR7+ CD56bright NK cells (%)
CCR7+ CD56dim NK cells (%)
D
CXCR6+ CD56bright NK cells (%)
CXCR6+ CD56dim NK cells (%)
E
Perforin+ CD56bright NK cells (%)
Perforin+ CD56dim NK cells (%)
F
Granzyme B+ CD56bright NK cells (%)
Granzyme B+ CD56dim NK cells (%)
G
CD57+ CD56bright NK cells (%)
CD57+ CD56dim NK cells (%)
H
KLRG1+ CD56bright NK cells (%)
KLRG1+ CD56dim NK cells (%)
I
CD16+ CD56bright NK cells (%)
CD16+ CD56dim NK cells (%)
Peg-IFNα therapy (EoT)
Sequential NUC
de novo NUC
9 months post Peg-IFNα (no further therapy)
