# Supplementary material for: Interferon Alpha Induces Sustained Changes in NK Cell Responsiveness to Hepatitis B Viral Load Suppression In Vivo
Source: PLoS Pathog. 2016 Aug 3;12(8):e1005788. doi: 10.1371/journal.ppat.1005788 (PMC4972354; doi:10.1371/journal.ppat.1005788)
Supplement: S1 Table — Numbers in brackets under headings; ALT, HBV DNA & HBsAg are mean values. * denotes ALT, HBV DNA & HBsAg at time of sequential NUC initiation. ** denotes ALT, HBV DNA & HBsAg at time of viral suppression. ^ denotes sustained HBsAg loss and § are patients sampled only at selected time-points on sequential NUC therapy. (PDF) [file ppat.1005788.s007.pdf]

Table S1 - Clinical parameters of sequential NUC therapy patients (Cohort 1).

	ALT* (IU/L) [138]	HBV DNA* (log_{10}IU/ml) [7.64]	HBsAg* (log_{10}IU/ml) [4.13]	ALT** (IU/L) [25]	HBV DNA** (log_{10}IU/ml) [1.30]	HBsAg** (log_{10}IU/ml) [3.20]	NUC analogue	HBsAg reduction on NUC	HBeAg seroconversion on NUC	CMV status	HLA-A2 status
Pt.2	153	8.53	4.53	36	1.30	3.43	Entecavir	Yes	No	Positive	Negative
Pt.3	118	7.71	4.06	40	1.30	3.47	Tenofovir	Yes	No	Positive	Negative
Pt.4	102	8.38	4.48	21	1.30	2.57	Tenofovir	Yes	Yes	Positive	Negative
Pt.6	202	8.42	4.13	19	1.30	4.13	Entecavir	No	No	Negative	Positive
Pt.8	81	6.98	3.37	15	1.30	3.88	Tenofovir	No	Yes	Positive	Positive
Pt.11	232	6.79	4.36	40	1.30	3.73	Tenofovir	Yes	Yes	Positive	Negative
Pt.13	204	3.37	3.89	20	1.30	0	Entecavir	Yes^	Yes	Positive	Positive
Pt.15	24	7.23	3.03	13	1.30	3.38	Entecavir	No	No	Positive	Negative
Pt.16	150	8.79	4.02	26	1.30	3.25	Tenofovir	Yes	No	Positive	Negative
Pt.19§	57	8.66	5.07	21	1.30	3.27	Entecavir	Yes	Yes	Positive	Positive
Pt.20§	138	8.17	4.02	21	1.30	3.83	Tenofovir	Yes	No	Positive	Negative
Pt.21§	152	8.68	4.54	18	1.30	3.37	Tenofovir	Yes	No	Positive	Positive
Pt.22§	99	8.17	3.83	27	1.30	2.87	Tenofovir	Yes	Yes	Positive	Negative
Pt.23§	232	7.05	4.52	33	1.30	3.81	Tenofovir	Yes	No	Positive	Negative

Numbers in brackets under headings; ALT, HBV DNA & HBsAg = mean values
* - denotes ALT, HBV DNA & HBsAg at time of sequential NUC initiation
** - denotes ALT, HBV DNA & HBsAg at time of viral suppression
^ - sustained HBsAg loss
§ - sampled only at selected time-points on sequential NUC therapy
