# Supplementary material for: Interferon Alpha Induces Sustained Changes in NK Cell Responsiveness to Hepatitis B Viral Load Suppression In Vivo
Source: PLoS Pathog. 2016 Aug 3;12(8):e1005788. doi: 10.1371/journal.ppat.1005788 (PMC4972354; doi:10.1371/journal.ppat.1005788)
Supplement: S2 Table — Numbers in brackets under headings; age is the median value for the cohort, ALT, HBV DNA & HBsAg are mean values. * denotes ALT, HBV DNA & HBsAg at time of de novo NUC initiation. ** denotes ALT, HBV DNA & HBsAg at time of viral suppression. ND–Test not done. (PDF) [file ppat.1005788.s008.pdf]

Table S2 - Clinical parameters of de novo NUC therapy patients (Cohort 2).

	Age [32]	Sex	Genotype	ALT* (IU/L) [95]	HBV DNA* (log_{10}IU/ml) [7.76]	HBsAg* (log_{10}IU/ml) [4.17]	ALT** (IU/L) [26]	HBV DNA** (log_{10}IU/ml) [1.30]	HBsAg** (log_{10}IU/ml) [3.98]	Fibrosis (Ishak)	NUC analogue	HBeAg sero conversion on NUC	CMV status	HLA-A2 status
Pt.24	40	M	ND	47	7.95	4.34	27	1.30	4.09	4	Tenofovir	No	Positive	Positive
Pt.25	29	M	ND	127	7.41	3.94	25	1.30	3.84	ND	Tenofovir	No	Positive	Positive
Pt.26	46	F	E	53	6.91	3.93	23	1.30	3.03	1	Entecavir	No	Positive	Negative
Pt.27	57	F	ND	65	6.90	3.37	10	1.30	3.25	1	Entecavir	No	Positive	Positive
Pt.28	37	M	C	353	8.99	4.20	32	1.30	4.26	5	Tenofovir	No	Positive	Negative
Pt.29	23	F	B	54	8.10	4.19	24	1.30	4.07	3	Tenofovir	No	Positive	Positive
Pt.30	34	F	B	72	7.98	4.39	37	1.30	4.22	1	Tenofovir	No	Positive	Positive
Pt.31	42	M	E	58	8.21	4.85	46	1.30	4.53	3	Tenofovir	No	Positive	Negative
Pt.32	59	F	D	60	8.02	4.39	32	1.30	4.48	3	Entecavir	No	Positive	Negative
Pt.33	29	M	D	111	7.80	4.06	12	1.30	3.99	ND	Entecavir	No	Positive	Negative
Pt.34	45	F	E	40	7.82	4.45	15	1.30	4.31	1	Tenofovir	No	Positive	Positive
Pt.35	42	M	E	95	7.11	3.95	31	1.30	3.74	ND	Tenofovir	No	Positive	Positive

Numbers in brackets under headings; age = median values; ALT, HBV DNA & HBsAg = mean values
* - denotes ALT, HBV DNA & HBsAg at time of de novo NUC initiation
** - denotes ALT, HBV DNA & HBsAg at time of viral suppression
ND – Test not done
