# Supplementary material for: Interferon Alpha Induces Sustained Changes in NK Cell Responsiveness to Hepatitis B Viral Load Suppression In Vivo
Source: PLoS Pathog. 2016 Aug 3;12(8):e1005788. doi: 10.1371/journal.ppat.1005788 (PMC4972354; doi:10.1371/journal.ppat.1005788)
Supplement: S3 Table — Numbers in brackets under headings; age is the median value for the cohort, ALT, HBV DNA & HBsAg are mean values. * denotes ALT, HBV DNA & HBsAg at baseline; prior to Peg-IFNα initiation. ** denotes ALT, HBV DNA & HBsAg at sampling time point (9–12 months post cessation of Peg-IFNα). # denotes sustained HBeAg seroconversion & HBV DNA <2000 IU/ml 6-months post cessation of Peg-IFNα, † indicates patients that refused sequential NUC therapy. (PDF) [file ppat.1005788.s009.pdf]

Table S3 - Clinical parameters of Peg-IFNα only therapy patients (Cohort 3).

	Age [31]	Sex	Genotype	ALT* (IU/L) [113]	HBV DNA* (log_{10}IU/ml) [7.08]	HBsAg* (log_{10}IU/m) [4.46]	ALT** (IU/L) [36]	HBV DNA** (log_{10}IU/ml) [4.39]	HBsAg** (log_{10}IU/ml) [4.07]	Fibrosis (Ishak)	CMV status	HLA-A2 status	Outcome
Pt.36	48	M	B	62	5.23	4.33	50	5.23	4.15	1	Positive	Positive	Non-Responder†
Pt.37	25	M	D	72	7.63	5.18	39	9.27	5.60	1	Positive	Positive	Non-Responder†
Pt.38	31	F	D	63	8.42	4.86	21	5.38	4.79	3	Positive	Negative	Non-Responder†
Pt.39	34	M	A	51	6.21	4.58	95	6.20	4.11	1	Positive	Negative	Non-Responder†
Pt.40	27	F	A	181	8.36	4.19	14	2.65	3.69	2	Positive	Positive	Responder#
Pt.41	30	F	C	381	7.10	4.91	13	3.12	3.68	1	Positive	Positive	Responder#
Pt.42	31	F	B	85	6.37	3.61	19	1.30	3.56	1	Positive	Negative	Responder#
Pt.43	31	M	C	65	7.12	3.81	23	2.20	3.15	4	Positive	Positive	Responder#
Pt.44	33	M	A	86	8.04	4.56	35	3.63	3.96	2	Positive	Negative	Non-Responder†
Pt.45	43	M	A	79	6.27	4.54	50	4.89	3.96	2	Positive	Negative	Non-Responder†

Numbers in brackets under headings; age=median values; ALT, HBV DNA & HBsAg mean values

* - denotes ALT, HBV DNA & HBsAg at baseline; prior to Peg-IFNα initiation.

** - denotes ALT, HBV DNA & HBsAg at sampling time point (12 months post cessation of Peg-IFNα)

# - Sustained HBeAg seroconversion & HBV DNA <2000 IU/ml 6-months post cessation of Peg-IFNα

† - Refused sequential NUC therapy
